# Polyphenolic Composition and In Vitro Antioxidant Activity of Red Grape Seeds as Byproducts of Short and Medium-Long Fermentative Macerations

**DOI:** 10.3390/foods9101451

**Published:** 2020-10-13

**Authors:** Antonella Bosso, Claudio Cassino, Silvia Motta, Loretta Panero, Christos Tsolakis, Massimo Guaita

**Affiliations:** 1 Consiglio per la Ricerca in Agricoltura e l’Analisi dell’Economia Agraria—Centro di Ricerca Viticoltura ed Enologia, via P. Micca 35, 14100 Asti, Italy; silvia.motta@crea.gov.it (S.M.); loretta.panero@crea.gov.it (L.P.); christos.tsolakis@crea.gov.it (C.T.); massimo.guaita@crea.gov.it (M.G.); 2 Dipartimento di Scienze e Innovazione Tecnologica, Università degli Studi del Piemonte Orientale, viale Teresa Michel 11, 15121 Alessandria, Italy; claudio.cassino@uniupo.it

**Keywords:** winemaking byproducts, grape seeds, polyphenols, antioxidant capacity

## Abstract

The polyphenolic composition and antioxidant activity of grape seeds, as byproducts of red winemaking, depend on various factors, such as grape cultivar, vintage effect, grape maturity and winemaking methods. In the present work, the influence of the maceration length on the polyphenolic and antioxidant characteristics of the seeds of four Italian red grape cultivars (‘Barbera’, ‘Grignolino’, ‘Nebbiolo’, and ‘Uvalino’), sampled from the fermentation tanks after short (two days) and medium-long (7–21 days) macerations, was studied with spectrophotometric methods, high-performance liquid chromatography (HPLC), and three different antioxidant assays (2,2′-azino-bis-3-ethylbenzothiazoline-6-sulfonic acid (ABTS), ferric reducing antioxidant power (FRAP) and 2,2 diphenyl-1-picrylhydrazyl (DPPH)). The total polyphenolic content (gallic acid equivalent (GAE)) of the seeds sampled after short macerations ranged between 24.5 and 60.1 mg/g dry weight (DW), and it dropped to 20.0–37.5 mg/g DW after medium-long macerations. The polyphenolic profile of the shortly macerated seeds was related to the varietal characteristics, while, after longer macerations, the influence of the maceration length prevailed on the cultivar effect. The multiple in vitro antioxidant activity tests (ABTS, FRAP and DPPH), although based on different mechanisms capable of highlighting behavioral differences between the different polyphenolic compounds, were highly correlated with each other and with the polyphenolic parameters; the qualitative differences between the matrices in the polyphenolic profile were probably less important than the quantitative differences in the polyphenolic content. The relations with the polyphenolic content were linear, except for the Efficient Concentration (EC_20_) parameter, whose relation was better described by a hyperbolic equation.

## 1. Introduction

Polyphenols can prevent the formation of free radicals in cellular tissues, providing a preventive action against various human pathologies, including cancer, cardiovascular and neurological diseases [1,2,3,4]. Recent studies highlighted the importance of polyphenols as components of nutritional supplements or functional foods [5,6], as food preservatives [7,8,9,10], and in cosmetic and pharmaceutical products [11].

The byproducts of the wine industry, in particular fresh (unfermented) and fermented grape pomace (GP), represent a potential source of natural polyphenolic compounds: generally, GP accounts for 20–30% of the initial weight of the grapes and can contain up to 70% of the original polyphenolic content of the grapes [12]. The stalks are normally removed before crushing/pressing the grapes for winemaking; therefore, GP is mainly composed of seeds and skins, which are the parts of the grape with the highest concentration in polyphenolic compounds [13]. The residual content of polyphenolic compounds in GP depends on grape variety, vintage and winemaking technique [14,15,16].

The polyphenolic composition and antioxidant activities of skins and seeds sampled from unfermented or fermented GP have been widely studied. Grape seeds are normally richer in polyphenolic compounds and have a higher antioxidant activity than grape skins, and the seeds from unfermented GP have higher polyphenols content and antioxidant activity than the seeds from fermented GP [15,17,18]. The polyphenolic content of the seeds from fermented GP depends on the grape cultivar, but above all on the winemaking conditions [18]. In the case of short macerations (for example, *rosé* wines, carbonic maceration wines, or red wines not suitable for aging), the losses in seed polyphenols during winemaking are probably limited so as not to preclude the interest for their exploitation as a source of antioxidant compounds. A winemaking technique which consists in removing part of the grape seeds from the fermenting tank during the first maceration days has been proposed for the red grape varieties characterized by seeds with high polyphenolic contents to obtain less astringent and smoother wines [19,20]. Seeds removal can be performed as soon as the cap of skins is completely raised by directly collecting the seeds deposited at the bottom of the tank (with conical bottom), or better during a pumpover or a *délestage* [21]: the seeds are separated from the must when it is pumped from the lower to the upper part of the same fermenting tank to soak the floating cap (pumpover), or when it is pumped to a support tank where it is oxygenated (*délestage*). These seeds, collected after short macerations, should generally have a high polyphenolic content and interesting antioxidant properties.

The fraction of extractable phenolics of the seeds accounts for 60–70% of the total extractable phenolics of the grapes. The phenol content of grape seeds can range from 5% to 8% by weight [22]. The seeds phenolics are mainly flavonoids: flavonols, flavanols, and more or less polymerized proanthocyanidins (condensed tannins). Grape seeds proanthocyanidins are composed by three different flavan-3-ols monomer subunits linked via 4–6 and 4–8 interflavan bonds: (+)-catechin, (−)-epicatechin, (−)-epicatechin-3-*O*-gallate, among which (−)-epicatechin is the main monomer subunit; the mean degree of polymerization (mDP) is about 10 units (for French cultivars) [23].

For the measurement of the antioxidant properties of foods, in particular fresh fruits and vegetables, different tests are used: DPPH (2,2 diphenyl-1-picrylhydrazyl), ABTS^+^ (2,2′-azino-bis-3ethylbenzothiazoline-6-sulfonic acid), FRAP (ferric reducing antioxidant power), ORAC (oxygen radical absorption capacity), CUPRAC (cupric reducing antioxidant capacity). The DPPH and ABTS tests are the most frequently used [24]. DPPH is a free radical that can accept an electron or a hydrogen atom from antioxidant donator molecules. The DPPH color changes from purple (maximum absorption band: 515–520 nm) to pale yellow (reduced form), so the antioxidant activity is assessed by spectrophotometry by measuring the variation over time of the color of the DPPH solution. The ABTS assay is based on the bluish-green cation radical ABTS^+^, which is generated through the reaction between ABTS and potassium persulfate. The generated radical has maximum absorption at 415, 645, 734 and 815 nm. Addition of antioxidants to the pre-formed radical cation reduces it back to ABTS. To determine the antioxidant activity, the decolorization at 734 nm is usually monitored [25,26]. The FRAP method has often been used together with DPPH and ABTS tests for the evaluation of the antioxidant power of grapes, wines and winemaking byproducts. This test evaluates the antioxidant activity by measuring the reduction of ferric tripyridyltriazine complex Fe(III)(TPTZ)_2_ to ferrous form Fe(II). FRAP is a simple direct test of antioxidant capacity. This method was initially developed [27] (Benzie and Strain, 1996) to assay plasma antioxidant capacity, but it can be used for plant extracts as well. The FRAP assay measures the absorbance change at 593 nm due to the formation of the blue colored Fe(II)-tripyridyltriazine complex from the colorless oxidized Fe(III) form by the action of electron donating antioxidants.

The present work aimed at characterizing the polyphenolic content and antioxidant capacity of grape seeds collected from the same fermentation tanks after a short maceration period (two days) and at the end of the fermentative maceration (7–21 days of maceration). The two different moments of the sampling wanted to simulate two different types of byproducts that can derive from the winemaking process: in the first case, seeds from GP racked off after short macerations or seeds removed early from the fermentation tanks; in the second case, seeds from GP racked off after medium-long macerations. In order to take into account the possible differences related to the raw material, the seeds of four Italian red grape cultivars (‘Barbera’, ‘Grignolino’, ‘Nebbiolo’ and ‘Uvalino’) were analyzed. These grape varieties are generally vinified with different winemaking processes: in order to reproduce the real winemaking conditions, we analyzed byproducts derived from large-scale vinifications in commercial wineries. The evaluation of the polyphenolic profile of the seeds concerned the total polyphenols and total flavonoids content, and the concentration and composition of condensed tannins (average size—mDP—and percentage of monomer subunits). The antioxidant capacity was assessed with DPPH, ABTS and FRAP tests.

This characterization of grape seeds as real winemaking byproducts was also aimed at suggesting the potential market value of this material, which is yearly generated in large amounts by the wine industry worldwide, due to the possibility of its exploitation as a source of natural polyphenols for many potential industrial applications, such as nutritional supplements in cattle feed and functional foods (nutraceuticals), food preservatives, cosmetics, and pharmaceutical or biomedical purposes.

## 2. Materials and Methods

### 2.1. Reagents

Phloroglucinol, L-ascorbic acid, sodium acetate, Folin–Ciocalteu reagent, hydrochloric acid 37%, formic acid 98%, and phosphoric acid 85% were purchased from Sigma-Aldrich Co., St. Louis, MO, USA; sodium carbonate and acetic acid 100% were purchased from Merck KGaA, Darmstadt, Germany. Commercial ethanol 95% *v/v* was used for the preparation of the extracts. Methanol high-performance liquid chromatography (HPLC) grade (VWR Chemicals, Radnor, PA, USA) and acetonitrile HPLC grade (Merck KGaA, Darmstadt, Germany) were used for the high-performance liquid chromatography (HPLC) analyses. The standards used for the phloroglucinolysis method were (+)-catechin, (−)-epicatechin, and (−)-epicatechin-3-*O*-gallate (Sigma Aldrich Co., St. Louis, MO, USA). C-18 solid-phase extraction cartridges (Sep-Pak tC18 1g) were purchased from Waters, Milford, MA, USA. Ultrapure water was used throughout the experiment (Milli-Q gradient A10—Millipore Corporation, Billerica, MA, USA).

### 2.2. Extraction of Polyphenols from Grape Seeds

The grape seeds of four different red grape cultivars widely cultivated in Piedmont (Italy), namely ‘Barbera’, ‘Grignolino’, ‘Nebbiolo’, and ‘Uvalino’, were sampled from the fermentation tanks at the respective wineries after 2 days of maceration and at the end of the fermentative maceration (racking off), after soft pressing (0.5 bar). The duration of the fermentative maceration was different: medium-short for ‘Barbera’ and ‘Grignolino’ (8 and 7 days, respectively) and long for ‘Nebbiolo’ and ‘Uvalino’ (21 and 18 days). The seeds sampled during the fermentative maceration (after 2 days of maceration) were collected during a pumpover or a *délestage*. The seeds were washed and dried at room temperature. After racking off, the sampling concerned the whole fermented pomace, which was air-dried in a ventilated oven (48 h, 35 °C) and then manually processed to separate the seeds from the grapes residues (skins and stalks). Dry seeds were ground with a coffee grinder (1 min), and the seed flours were extracted with a hydroalcoholic solution, according to a previous work [28]: 0.6 g of seed flour was extracted with 6 mL of ethanol/water (1:1) in a 15 mL Corex tube, vortexed for 30 s, sonicated for 20 min (50 W, 48 kHz ± 10%), and then again vortexed for 30 s. The sample was centrifuged at 18 °C for 20 min at 2880× *g* (Centrifuge 5810 R Eppendorf—Hamburg, Germany), then the supernatant (5 mL) was separated from the solid residue, and vacuum dried at 35 °C with a Genevac evaporator (EZ-2, Genevac©, Ipswich, UK). All extractions were performed in triplicate.

### 2.3. Spectrophotometric Analysis

The dry residue of the extracts was re-dissolved in 3 mL methanol for the subsequent analysis of polyphenols. Total flavonoids and total polyphenols were determined by spectrophotometry [29]:

- Total flavonoids: the extract was diluted 50-fold with hydrochloric ethanol (ethanol/H_2_O/HCl; 70:30:1) and the absorbance at 280 nm was measured. The results were expressed as (+)-catechin equivalents.
- Total polyphenols: 1 mL extract was diluted 20-fold with water, and 1 mL of diluted sample was added to 1 mL of Folin–Ciocalteu reagent, basified with 4 mL of sodium carbonate, and filled up to 20 mL. After 90 min, the absorbance at 750 nm was measured against a blank. The results were expressed as mg of gallic acid equivalents (GAEs)/g dry weight (DW) of seed flour.

### 2.4. Characterization of Condensed Tannins (Phloroglucinolysis) and Monomer Flavan-3-Ols by HPLC

Phloroglucinolysis is the acid-catalyzed cleavage of the condensed tannins (proanthocyanidins) in the presence of excess phloroglucinol as nucleophile molecule, and the reaction products are analyzed with HPLC [30]. The operating protocol was as reported by [31], with some modifications. The dry residue of the extracts was re-dissolved in 2.5 mL of 2% aqueous acetic acid and 1.5 mL was applied on a C-18 SPE cartridge. Sugars and organic acids were eluted with 5 mL of 2% aqueous acetic acid, the phenolic compounds were recovered with 8 mL of methanol. The methanolic sample was dried under vacuum, re-dissolved in 400 μL of the reactive solution (250 mg phloroglucinol + 50 mg ascorbic acid in 5 mL methanol/HCl 98:2) and heated for 25 min at 50 °C. The reaction was quenched with 400 μL of aqueous 200 mM sodium acetate. The sample was filtered with a 0.45 μm polypropylene syringe filter (Whatman Inc., Piscataway, NJ, USA) directly into the HPLC vial. The injection volume was 10 μL. Samples were analyzed with an Agilent 1200 HPLC system (Agilent Technologies, Palo Alto, CA, USA) with a reversed-phase Atlantis T3 dC18 column (5 μm packing, 250 × 2.1 mm i.d., Waters, Milford, MA, USA) protected by a guard column of the same material (10 × 2.1 mm i.d., Waters, Milford, MA, USA). The HPLC operating conditions were the same as those reported by [31]. The monomers (terminal and extension subunits, the latter as phloroglucinol adducts) were detected by a DAD detector (Agilent G1315D), identified by comparison with the retention times and quantified from peak areas at 280 nm with the Chemstation software (Agilent Technologies, Palo Alto, CA, USA) using external calibration with known concentrations of (+)-catechin, (−)-epicatechin and (−)-epicatechin-3-*O*-gallate from Sigma (Saint Louis, MO, USA). The response factors of the extension subunits were calculated using the response factor of (+)-catechin and considering the respective values of the molar extinction coefficients [30]. The molar concentration of the subunits was calculated from their molar absorptivities as reported by [30], and expressed as molar percentage. The mean degree of polymerization (mDP) was calculated as follows: the sum of the terminal and extension subunits (in moles) was divided by the sum of the terminal subunits (in mols). The content in total condensed tannins, expressed in mg per g DW of seed flour, was determined as the sum of all subunits. 

Monomer flavan-3-ols—(+)-catechin and (−)-epicatechin—were determined with the same HPLC method used for the phloroglucinolysis method, excluding the reaction with phloroglucinol, as reported above.

### 2.5. Antioxidant Activity Tests

#### 2.5.1. DPPH Test

For the DPPH test, 135 μM DPPH solution in methanol was used as standard DPPH reagent (2,2-diphenyl-1-picrylhydrazyl). For antioxidant activity measurements, 0.56 mL of seed extract (corresponding to 0.067 g of seed flour) was vacuum dried, treated with 4 mL of hydroalcoholic buffer (25% ethanol, tartaric acid 2 g/L, pH 3.2) and placed in an ultrasonic bath for 10 min to promote complete dissolution. A total of 40 μL of this solution was added to 3.96 mL of the DPPH reagent. Absorbance was measured at 517 nm after 30 min reaction at room temperature. The measurements were repeated on three different polyphenols extract aliquots and each sample was measured in triplicate. Percentage inhibition (I%) was calculated from absorbance using the formula I% = (A_0_ − A)/A_0_ × 100, where A_0_ is the absorbance of the control and A is the absorbance of the sample. The percentage inhibition of the sample was then compared with a standard curve obtained from the corresponding readings of ascorbic acid (DPPH_AAE_) or Trolox (DPPH_TE_). The results are reported as Trolox equivalent (mmol TE/100 g of seeds flour (DW)) and as ascorbic acid equivalent (mg AAE/g DW).

A second DPPH test was performed according to the method proposed by [32]. A concentrated stock solution was prepared with 6–7 mg of DPPH reagent in 25 mL of methanol and placed in an ultrasonic bath for 10 min to promote complete dissolution. The solution to be used for the test was obtained by diluting 10-fold the stock solution, to an approximate DPPH concentration of 24–30 mg/L. The exact concentration of this DPPH solution was calculated based on the absorbance value at 515 nm, with the calibration curve:

y = 0.0254 [DPPH] + 0.036; R^2^ = 0.9994

where [DPPH] is the concentration of DPPH in mg/L and y is the absorbance of the solution at time zero.

The dry residue of the seed extracts was re-dissolved in 3 mL methanol, and then it was diluted: based on the total polyphenols concentration (GAE) of the methanolic extract, two dilutions were chosen, such as to determine a percentage DPPH inhibition of about 20%, and included between 20% and 40%. A total of 2970 μL of DPPH reagent was placed in a 1 cm o.p. cuvette, then 30 μL of diluted extract was added. Absorbance was measured after 240 min of storage at room temperature in the dark. The data were expressed as Efficient Concentration (EC_20_), which indicates the quantity in mg of seed flour (DW) necessary to determine a 20% inhibition to 1 mg of DPPH.

#### 2.5.2. ABTS Test

ABTS^+^ (2,2′-azino-bis(3-ethylbenzothiazoline-6-sulfonate)) was prepared by mixing an ABTS stock solution (7 mM in water) with 2.45 mM potassium persulfate. This mixture was allowed to stand overnight at room temperature in the dark. A 400 μL aliquot of the obtained solution was then diluted to 25 mL with methanol and used as standard ABTS reagent. For antioxidant activity measurements, 0.56 mL of seed extract (corresponding to 0.067 g of seed flour) was vacuum dried, treated with 8 mL of hydroalcoholic buffer (25% ethanol, tartaric acid 2 g/L, pH 3.2) and placed in an ultrasonic bath for 10 min to promote complete dissolution. A 20 μL aliquot of this solution was added to 3.98 mL of the ABTS reagent. Absorbance was measured at 744 nm after a 30 min reaction at room temperature. The measurements were repeated on three different polyphenols extract aliquots and each sample was measured in triplicate. Percentage inhibition (I%) was calculated as reported for DPPH. The percentage inhibition of the sample was then compared with a standard curve made from the corresponding readings of ascorbic acid (ABTS_AAE_) or Trolox (ABTS_TE_). The results are reported as Trolox equivalent (TE, mmol/100 g DW) and as ascorbic acid equivalent (AAE, mg/g DW).

#### 2.5.3. FRAP Test

The FRAP reagent was prepared by mixing 0.3 M acetate buffer (pH 3.6), 10 mM TPTZ ((2,4,6-tris(2-pyridyl)-s-triazine)), and 20 mM ferric chloride (10:1:1 *v*:*v*:*v*). For antioxidant activity assays, 0.56 mL of seed extract (corresponding to 0.067 g of seed flour) was vacuum dried, treated with 8 mL of hydroalcoholic buffer (25% ethanol, tartaric acid 2 g/L, pH 3.2) and placed in an ultrasonic bath for 10 min to promote complete dissolution. A 20 μL aliquot of this solution was added to 3.98 mL of the FRAP reagent. Absorbance was measured at 596 nm after a 30 min reaction at room temperature. The absorbance of each sample was compared with those obtained from the standard curve made from Trolox (FRAP_TE_) or ascorbic acid (FRAP_AAE_). The Fe^2+^ concentration in the sample was calculated from the absorbance reading at 596 nm using 22,230 M^−1^cm^−1^ as a molar extinction coefficient for the Fe(II)(TPTZ)_2_ complex [33]. Data are reported as Trolox equivalent (TE, mmol/100 g DW), ascorbic acid equivalent (AAE, mg/g DW) and mmol Fe^2+^/100 g DW.

### 2.6. Statistical Analysis

All data were processed with Analysis of Variance (ANOVA) and Tukey’s test. The correlation analysis was also applied to study the relationships between the total polyphenols content (GAE) and the parameters ABTS, DPPH, FRAP and EC_20_. XLSTAT 2017 (Data Analysis and Statistical Solution for Microsoft 250 Excel. Addinsoft, Paris, France, 2017) was used.

## 3. Results and Discussion

### 3.1. Seeds Sampled after Two Days of Maceration

Table 1 shows the polyphenolic composition of the extracts obtained from the seeds sampled after two days of maceration, when the alcohol content was about 5% v/v. Significant differences were observed for almost all the chemical parameters analyzed.

The total polyphenols (GAE) and total flavonoids content ranked significantly in the order ‘Barbera’ < ‘Nebbiolo’ < ‘Grignolino’ < ‘Uvalino’. ‘Barbera’ seeds had the significantly lowest GAE value (24.5 mg/g DW), and ‘Uvalino’ the significantly highest (60.1 mg/g DW). The same trend was observed for the condensed tannins content. After two days of maceration, the ranking of the cultivars—limited to ‘Barbera’, ‘Nebbiolo’ and ‘Uvalino’—based in particular on the condensed tannins content of the seeds, was similar to that observed in a previous work on the same grape varieties [18] and to the results reported in other works concerning the comparison between ‘Barbera’ and ‘Nebbiolo’ grapes as regards the condensed tannins content (proanthocyanidins) [34,35] and between ‘Barbera’, ‘Grignolino’ and ‘Nebbiolo’ grapes regarding the content of total polyphenols, total flavonoids and condensed tannins (proanthocyanidins) [36]. To our knowledge, no other works in the literature, besides [18], concern the polyphenolic characterization of ‘Uvalino’ grape seeds.

Significant differences between cultivars were also observed in the average size (mDP) and percentage monomer composition of the condensed tannins (Table 1). All monomer units typical of grape seed tannins were identified and quantified: (+)-catechin (C), (−)-epicatechin (EC) and (−)-epicatechin-3-*O*-gallate (ECG), as both terminal units and extension units bond to phloroglucinol. Moreover, (−)-epigallocatechin (EGC), typically found only in skin tannins [23], was present in traces among the extension units, probably due to the adsorption of skin tannins on the seed’s surface during maceration.

The main differences between cultivars concerned the galloylation degree (G%) and the percentage content of C and EC as terminal monomer units. The condensed tannins of ‘Barbera’ and ‘Nebbiolo’ had a significantly higher G% (19–20%) than ‘Uvalino’ and ‘Grignolino’ tannins (18 and 15%, respectively); ‘Grignolino’ tannins were significantly distinguished from the others by the lowest G%. The condensed tannins of ‘Barbera’ and ‘Nebbiolo’ had also the highest average size (mDP = 4) and the lowest percentage of terminal monomer units.

Although C and EC were the prevailing sub-units of the polymeric chains of tannins (up to 80%), the content in C and EC as monomer flavan-3-ols was very low (3.7–10.2% w/w) compared to condensed tannins. Among monomer flavan-3-ols, C was more abundant than EC, and the C/EC ratio varied from 1.1 (‘Barbera’) to 2.3 (‘Nebbiolo’). Significant differences were observed between the cultivars: ‘Barbera’ seeds had the significantly lowest content in C and EC, while ‘Nebbiolo’ seeds had the significantly highest content in C.

Since no data are available in the literature on the composition of grape seeds sampled after short macerations, our results were compared with the values observed for the unfermented grape seeds of the respective cultivars, when present in the literature.

The total content in polyphenolic compounds of ‘Barbera’, ‘Nebbiolo’ and ‘Uvalino’ seeds, sampled after two days of maceration, was lower than the values reported in our previous work [18] for the unfermented seeds of the same three grapes cultivars. The differences in total polyphenols content (GAE) between the unfermented seeds and those determined after two days of maceration varied from −45% for ‘Uvalino’ to −60 and −70% for ‘Nebbiolo’ and ‘Barbera’. The percentage losses were even greater for the condensed tannins content, but more similar for the three cultivars (ranging between −68 and −75%). Since it was not the same raw material and the same vintage of the cited work, it is not possible to evaluate the extent of the releases of polyphenols that occurred during the first days of maceration. These results are however interesting from an oenological point of view and, if confirmed, they could highlight how the grape seeds start to contribute to the polyphenolic content of wines since the first days of maceration, when the alcohol content in the medium is still relatively low. The same decrease was observed for the content in total flavonoids and condensed tannins. On the contrary, the average size (mDP) and the galloylation degree (G%) of the condensed tannins of the seeds sampled after two days of maceration were similar to the respective values of the unfermented seeds of the corresponding cultivars, as reported in the cited work [18].

The content in polyphenolic compounds (GAE) of grape seeds varies according to the cultivar and the vintage. The GAE values of the seeds sampled after two days of maceration were medium-high when compared with those reported below, found in the literature, and referred to seeds from unfermented grapes of other cultivars. In a work on some French grape cultivars (‘Grenache’, ‘Syrah’, ‘Carignan noir’, ‘Mourvèdre’, ‘Counoise’ and ‘Alicante Bouchet’), [15] reported GAE values for the seeds ranging from 37 to 50 mg/g DW in 2009 and from 59 to 89 mg/g DW in 2010; [37] reported average GAE concentrations in grape seeds equal to 114 mg/g DW for ‘Pinot Gris’ and 16 mg/g DW for ‘Regent’ (interspecific hybrid); [38] found values ranging from 8 to 33 mg/g in seeds of native Greek *Vitis vinifera* cultivars; [39] reported average GAE concentrations of 16–52 mg/g in seeds of red grape cultivars grown in Central Italy.

### 3.2. Seeds Sampled at Racking off

Table 2 shows the polyphenolic composition of the extracts obtained from the seeds sampled after racking off. Significant differences between the four cultivars were observed for all the chemical parameters analyzed. Regarding the polyphenolic content, the four cultivars ranked differently compared to the sampling after two days of maceration; in many cases, the differences between cultivars disappeared. In particular, at racking off the ‘Grignolino’ seeds were significantly different from those of other cultivars (similar to each other) for the highest content in total polyphenols, total flavonoids and condensed tannins.

The data were compared with the values reported in the literature. The total polyphenols content (GAE) of the seeds at racking off was 20–37.5 mg/g DW, similar to the values reported by [15] for fermented seeds of French cultivars, whose GAE content varied between 12.6–35.3 mg/g DW (2009 vintage) and 33–40.8 mg/g DW (2010 vintage). Average values higher than 85.8 mg/g DW were reported by [40] for fermented seeds of ‘Negroamaro’ cultivar.

### 3.3. Comparison between the Two Samplings

The data relating to the two samplings (2 days of maceration and racking off) were compared with complete 2-factor ANOVA (factors: cultivar and sampling time) (Table 3). The polyphenolic content of the seeds decreased significantly with the maceration length, and the composition of condensed tannins changed.

The extent of the losses of polyphenols varied according to the cultivar and the maceration length, as it is evidenced by highly significant interactions between the “cultivar” and “sampling time” factors. In particular, the losses of GAE were −18, −21, −44 and −62%, while those in condensed tannins were −14, −25, −42 and −51%, respectively for ‘Barbera’, ‘Grignolino’, ‘Nebbiolo’ and ‘Uvalino’. These results were probably due to the differences in maceration length, according to the specific winemaking protocols (varietal oenology): medium for ‘Barbera’ and ‘Grignolino’ and long for ‘Nebbiolo’ and ‘Uvalino’ (see Section 2).

Regarding the composition of condensed tannins (Table 2), at racking off the mean degree of polymerization (mDP) increased for all cultivars compared to the first sampling, especially for ‘Uvalino’, and variations were observed in the percentage weight of the monomer units. In particular, the percentage content of C proportionally decreased as terminal unit, and increased as extension unit. An increase was also observed in the EGC content, a molecule that is generally present only in the skins, which is probably adsorbed on the surface of the seeds during maceration, though in low concentrations. No significant differences were observed in the galloylation degree of the condensed tannins (Table 3). Conversely, the flavan-3-ols content dropped significantly from the first to the second sampling (Table 3). The largest losses concerned the cultivars subjected to long macerations: −70% for ‘Nebbiolo’ seeds and −78% for ‘Uvalino’ seeds. Referring to each cultivar, the percentage decreases in the content of C and EC were similar (Table 2). The influence of winemaking conditions (duration of maceration) on the polyphenolic composition of the seeds at racking off was more important than the varietal compositional differences. These results were in agreement with a previous work [18].

### 3.4. Antioxidant Activity

The antioxidant activity of the grape seed extracts was measured with the ABTS, DPPH and FRAP tests (Table 4). The results are expressed as mg of ascorbic acid equivalent (AAE) per 1 g of seed flour (1 g DW) and as mmols of Trolox equivalent (TE) per 100 g DW. Moreover, the results of DPPH test are also expressed as EC_20_, according to the method proposed by [32], and the FRAP results are also expressed as mmols Fe^2+^/100 g DW.

At the first sampling (after two days of maceration, Table 4a), the ranking of the cultivars based on the response of the seed extracts to the tests was the same as for the polyphenolic content: the results of ABTS, DPPH and FRAP tests increased in the order ‘Barbera’ < ‘Nebbiolo’ < ‘Grignolino’ < ‘Uvalino’. According to ABTS and FRAP tests, the seeds of the four cultivars resulted significantly different from one another, while, according to the DPPH test, ‘Nebbiolo’ and ‘Grignolino’ seeds resulted similar, as observed for the GAE parameter. Regarding the EC_20_ parameter, ‘Barbera’ (highest values) and ‘Nebbiolo’ seeds (intermediate values) were significantly different from one another and from ‘Grignolino’ and ‘Uvalino’ seeds (lowest values), which were similar to each other: unlike the other parameters, EC_20_ did not discriminate from one another the samples with the highest polyphenol contents, i.e., those with the lowest E_20_ values.

At racking off (Table 4b), ‘Grignolino’ seeds had the significantly highest values for the ABTS, FRAP and DPPH parameters. The ABTS test did not distinguish from one another the other three cultivars; the same result was observed for the polyphenolic content (total polyphenols, total flavonoids and condensed tannins, Table 2). On the contrary, the FRAP test distinguished ‘Uvalino’ (higher values) from ‘Barbera’ and ‘Nebbiolo’, similar to each other; the DPPH test distinguished ‘Barbera’ (lowest values) from ‘Nebbiolo’ and ‘Uvalino’, similar to each other; EC_20_ distinguished ‘Grignolino’ (lowest values) from ‘Nebbiolo’ and ‘Barbera’, while ‘Uvalino’ (intermediate values) was not significantly different from the other cultivars.

The literature on the methods for the determination of the antioxidant properties of grapes, wines and winemaking byproducts often report different analytical protocols (quantity of sample, doses of reagents, reaction time, etc.) and ways of expressing the results. To compare our results to the values reported in the literature, the data regarding the antioxidant activity of the seed extracts were expressed in different ways (ascorbic acid equivalents, Trolox equivalents for ABTS, FRAP and DPPH, and mmols of Fe^2+^ for the FRAP test), and the DPPH test was performed according to two different methods. 

The ABTS values expressed as mg AAE/g DW related to ‘Barbera’, ‘Nebbiolo’ and ‘Uvalino’ seeds at the first sampling (Table 4a), were compared to the corresponding values determined for the same three cultivars in a previous work [18]: the average ABTS values for grape seeds were higher and ranged from 117.6 (‘Barbera’) to 185.5 mg AAE/g DW (‘Uvalino’), proportionally to the higher total polyphenols contents (GAE). Processing together the data collected during the two works, a close correlation (r = 0.983) was observed between the ABTS values determined for the seeds of the three cultivars and the corresponding total polyphenols contents (GAE). At the first sampling (Table 4a), the FRAP values ranged between 22.4 and 55.6 mmols TE/100 g DW, and the ABTS values between 19.2 and 32.8 mmols TE/100 g DW. The FRAP values were comparable to those reported by [37] for the seeds of some *Vitis vinifera* cultivars and interspecific hybrids, while the ABTS values were similar for some cultivars (e.g., ‘Seyval blanc’ and ‘Freiminer’) and lower for others (e.g., ‘Pinot gris’ and ‘Rondo’), as reported in the same work [37].

The ABTS and DPPH values determined at racking off (Table 4b) and expressed as TE were compared with the results reported by [15] for seeds from French red grapes pomace with similar total polyphenols contents (GAE). The ABTS and DPPH values determined in our work were lower than those reported by the aforementioned authors (40.4–60.3 mmols TE/100 g DW for ABTS and 26.3–53.5 mmols TE/100 g DW for DPPH). The differences in antioxidant properties could be due to qualitative differences in the polyphenolic composition of the extracts. For example, our seed extracts had a lower galloylation degree (G%) than the extracts analyzed by [15]: the gallic acid esterification was observed to increase the free radical scavenging capacity of the dimer procyanidins [22]. On the contrary, the FRAP values (expressed as Fe^2+^) determined by [15] were lower than the values determined for our seed extracts. The discrepancy in results between the ABTS and DPPH tests and the FRAP test observed for seed samples of different origins and with a similar total polyphenol content is not contradictory. These tests, in fact, measure different antioxidant properties of the matrices, so the results can be affected by the matrix effect [14].

The EC_20_ parameter was calculated according to the method proposed by [32] for wines and oenological byproducts, as described in Materials and Methods. The authors proposed this method to solve some problems they noticed in performing the DPPH test for complex matrices, such as wines and other oenological byproducts. In particular, the authors noticed that, unlike with pure substances (for example, catechin, ascorbic acid), in the case of complex matrices, the percentage DPPH inhibition grew according to a nonlinear trend with the increase of the polyphenolic concentration of the analyzed sample (ratio between the polyphenolic concentration and the DPPH concentration). On the contrary, for all the studied matrices, the relationship between polyphenolic concentration and percentage DPPH inhibition became linear when operating with low sample concentrations, such as to determine a percentage DPPH inhibition lower than 40%. Hence, the proposal to work with more diluted samples and to calculate a new parameter, EC_20_, which measures the quantity of product (mg of dry matter or mL of wine) capable of inhibiting by 20% the free radical activity of DPPH, instead of 50% as previously proposed by other authors [41]. Recently, other authors [24] verified the absence of linearity in the relation between polyphenolic concentration and percentage inhibition of radical activity (% I) used to calculate the EC_50_ parameter, and proposed new algorithms that better fit the relationship between the two parameters. At present, to our knowledge, examples of EC_20_ calculated for grape seeds derived from pomace are not reported in the literature. Conversely, in some works, the parameter EC_50_ was calculated (50% inhibition of DPPH activity). The authors of [39] reported EC_50_ values ranging between 0.62 and 1.25 for the seeds of some grape cultivars grown in central Italy; [42] reported EC_50_ values ranging between 0.24 and 0.92 for the seeds of nine Hellenic native and international Vitis vinifera varieties cultivated in Greece. From the extrapolation of the data from EC_20_ to EC_50_, the values determined in the present work become of the same order of magnitude as those determined by the aforementioned authors, considering also that our data were not referred to unfermented grape seeds, but to seeds that were subjected to macerations of different duration.

**Figure 1:**
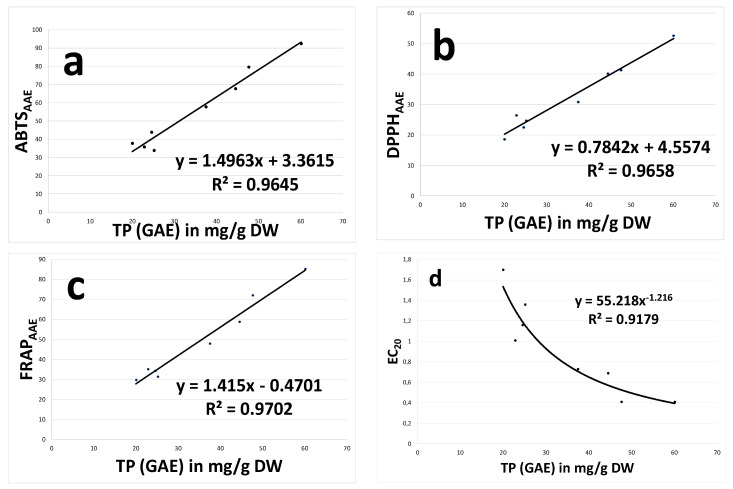
Representation of the relations between the total polyphenols content (GAE) and the parameters ABTS (**a**), DPPH (**b**), FRAP (**c**) and EC_20_ (**d**). ABTS, 2,2′-azino-bis-3-ethylbenzothiazoline-6-sulfonic acid; FRAP, ferric reducing antioxidant power; DPPH, 2,2 diphenyl-1-picrylhydrazyl; EC_20_, Efficient Concentration.

Table 5 shows the correlation matrix calculated with the variables related to the polyphenolic composition (total polyphenols, total flavonoids and condensed tannins) and the results of the antioxidant tests performed for the seeds of both samplings (two days of maceration and racking off). High correlation degrees were observed between all parameters. The values of the correlation coefficients (r) between the ABTS, FRAP and DPPH parameters and the total polyphenols content (TP) were, respectively, 0.982, 0.985 and 0.983. The presence of highly significant correlations with the other parameters was also observed for EC_20_, but the r values were slightly lower, for example, the r value of the correlation between EC_20_ and GAE was −0.908 and the negative sign expresses the inverse correlation, because the EC_20_ values decrease with the increase in the antioxidant power of the sample. Furthermore, while the relations between the other antioxidant tests and the parameters related to the phenolic content were better fitted by a linear equation (Figure 1a–c), in the case of EC_20_ the relation with the phenolic content was better fitted by a hyperbolic equation (Figure 1d). A similar relation between the EC_50_ parameter and the polyphenolic content (GAE) was reported in the work of [38].

In our previous work [18], we verified the presence of a strong linear correlation between the ABTS results and the parameters related to the polyphenolic content of the seed extracts (GAE, total flavonoids, proanthocyanidins, condensed tannins); the same was not observed between ABTS results and total polyphenols (GAE) of the skin extracts. Other authors [14] observed significant correlations between DPPH and polyphenolic content (particularly total flavonoids and flavanols) of polyphenolic extracts from oenological byproducts. The authors of [38] observed a highly significant linear correlation (r = 0.984) between total polyphenols and the FRAP index of polyphenolic extracts of grapes, wines and oenological byproducts.

In our work, all tests used were highly correlated with each other and with the values of the polyphenolic contents, but, in our opinion, this result cannot be generalized since it depends on the matrix composition of the studied samples. Generally, the use of multiple tests is recommended for the evaluation of the antioxidant properties of a complex matrix, and even better if the tests are based on different mechanisms, precisely in order to take into account the behavioral differences between the different polyphenolic compounds that compose the matrix. In our specific case, among the three tests used, the FRAP test measures the reducing capacity of the molecules, while the ABTS and DPPH tests measure the capacity to donate a hydrogen or an electron. Despite the different mechanisms, when used to measure the antioxidant activity of seed flour, these tests were strongly correlated with each other, and this could depend, as suggested by [14], on the fact that our matrices (seeds from different cultivars sampled at different moments of the winemaking cycle) were composed of similar polyphenolic compounds; in our case, the main differences between the seed samples concerned the quantity rather than the quality of the polyphenolic compounds.

Regarding the composition of the condensed tannins (flavans) of the seeds, the differences between samples were modest: mDP varied between 3.2 and 4.0 units at the first sampling (after two days of maceration) and between 4.2 and 5.0 units at the second sampling (racking off). In all cases, the prevailing monomer unit was EC (83.8–86.3% first sampling, 77.6–86.3% second sampling), and the average galloylation degree was 15.2–20.3% at the first sampling and 16.5–21.7% at the second sampling. In this specific case, the high multicollinearity between the variables suggests that the polyphenolic characterization of the seeds can be easily performed with a reduced number of parameters, and even with only one of these. The extension of the study to the seeds of a higher number of cultivars and different vintages will allow for the confirmation of this possibility.

## 4. Conclusions

The polyphenolic composition of the seeds of four Italian red grape cultivars (‘Barbera’, ‘Grignolino’, ‘Nebbiolo’ and ‘Uvalino’) was determined after a short maceration period (two days) and at racking off (after 7–21 days of maceration) in order to simulate the differences between two possible oenological byproducts. The polyphenolic profile of the seeds sampled after two days of fermentative maceration was related to the varietal characteristics, while, at racking off, it was mainly affected by the maceration conditions. The greatest losses in polyphenolic compounds (total flavonoids, total polyphenols and condensed tannins) during the fermentation maceration were observed for ‘Nebbiolo’ and ‘Uvalino’ seeds, the cultivars that were subjected to longer maceration times. After racking off, ‘Grignolino’ seeds had the significantly highest content in total polyphenols, total flavonoids and condensed tannins, and the highest antioxidant activity. As regards the qualitative aspects, minor differences were observed between cultivars in the composition of condensed tannins, in particular for the average size of the polymeric chains (mDP) and for the percentage weight of the various monomer units. The qualitative differences observed between the cultivars and between the sampling times were, on the whole, rather modest; under these conditions, the tests used to evaluate the antioxidant properties of the grape seeds (DPPH, ABTS and FRAP), although being based on different mechanisms, were all highly correlated with each other and with the parameters describing the polyphenolic composition.

During the present work, we observed that the seeds sampled from the fermentation tank after short macerations (two days) still maintained a high polyphenolic content, a condensed tannin composition similar to that of the cultivar of origin, as observed in previous works, and a high antioxidant activity. These seeds represent a precious source of polyphenolic compounds, which can be used on an industrial level: the data collected during the work provide a preliminary characterization of the polyphenolic profile and of the antioxidant properties of these winemaking byproducts.

## Figures and Tables

**Table 1:** Polyphenolic composition of the grape seeds sampled from the fermentation tanks after 2 days of maceration. Analysis of Variance (ANOVA) and Tukey’s test results. mDP = mean degree of polymerization; G% = percentage of galloylation; EGC = (−)-epigallocatechin; C = (+)-catechin; EC = (−)-epicatechin; ECG = (−)-epicatechin-3-*O*-gallate.

2 Days of Maceration		Barbera	Grignolino	Nebbiolo	Uvalino	F_cv_	Sig
Total flavonoids (mg/g DW)		38.8 ± 0.5 a ^1^	93.1 ± 1.4 ab	70.8 ± 2.0 a	137.4 ± 3.5 b	15	* ^2^
Total polyphenols (GAE) (mg/g DW)		24.5 ± 0.4 a	47.6 ± 1.4 b	44.5 ± 3.4 b	60.1 ± 1.0 c	120	***
mDP		4.0 ± 0.03 b	3.2 ± 0.04 a	4.0 ± 0.29 b	3.5 ± 0.07 ab	14	*
G%		20.3 ± 0.05 c	15.2 ± 0.05 a	19.4 ± 0.44 c	17.8 ± 0.01 b	196	***
Condensed tannins (mg/g DW)		14.7 ± 0.3 a	24.1 ± 1.1 bc	22.4 ± 0.2 b	26.6 ± 0.5 c	126	***
Extention units (%)	EGC	0.36 ± 0.00 b	0.22 ± 0.00 a	0.36 ± 0.03 b	0.23 ± 0.00 a	17	*
	C	3.57 ± 0.40 b	2.63 ± 0.15 a	3.24 ± 0.12 ab	3.09 ± 0.02 a	6.3	*
	EC	55.6 ± 0.8	55.4 ± 0.5	58.3 ± 1.6	57.4 ± 0.5	4.2	ns
	ECG	16.0 ± 0.6 c	10.2 ± 0.1 a	12.9 ± 0.4 b	10.3 ± 0.1 b	121	***
Terminal units (%)	C	10.1 ± 0.3 a	13.6 ± 0.2 b	10.4 ± 1.2 a	11.2 ± 0.1 ab	12	*
	EC	9.8 ± 0.7 a	12.9 ± 0.2 b	8.4 ± 0.7 a	10.2 ± 0.4 a	26	**
	ECG	4.6 ± 0.04 a	5.0 ± 0.02 b	6.4 ± 0.08 c	7.5 ± 0.08 d	1048	***
Flavan-3-ols (mg/g DW)	C	0.28 ± 0.00 a	1.14 ± 0.17 bc	1.59 ± 0.10 c	0.98 ± 0.02 b	30	**
	EC	0.26 ± 0.01 a	0.89 ± 0.13 b	0.69 ± 0.02 b	0.72 ± 0.02 b	15	*

^1^ Different letters along the line discriminate the cultivars significantly different from one another (*p* < 0.05, Tukey’s test). ^2^ Significance: *, **, *** and ns represent significance at *p* ≤ 0.05, 0.01, 0.001 and not significant, respectively. DW, dry weight. GAE, gallic acid equivalent.

**Table 2:** Polyphenolic composition of the grape seeds sampled from the fermented pomace after racking off. ANOVA and Tukey’s test results. mDP = mean degree of polymerization; G% = percentage of galloylation; EGC = (−)-epigallocatechin; C = (+)-catechin; EC = (−)-epicatechin; ECG = (−)-epicatechin-3-*O*-gallate.

Racking off		Barbera	Grignolino	Nebbiolo	Uvalino	F_cv_	Sig
Total flavonoids (mg/g DW)		30.7 ± 0.5 a ^1^	64.9 ± 3.5 b	35.4 ± 0.6 a	31.8 ± 0.2 a	167	*** ^2^
Total polyphenols (GAE) (mg/g DW)		20.0 ± 0.1 a	37.5 ± 2.8 b	25.1 ± 0.2 a	22.8 ± 0.3 a	60	***
mDP		4.8 ± 0.02 b	4.1 ± 0.02 a	4.2 ± 0.04 a	5.0 b ± 0.02	50	***
G%		21.7 ± 0.2 c	16.5 ± 0.2 a	17.9 ± 0.4 b	17.5 ± 0.3 ab	144	***
Condensed tannins (mg/g DW)		12.6 ± 0.8 a	18.0 ± 1.8 b	13.0 ± 0.5 a	13.1 ± 0.9 a	11	*
Extention units (%)	EGC	0.47 ± 0.01 c	0.27 ± 0.04 a	0.45 ± 0.01 bc	0.33 ± 0.04 ab	20	**
	C	9.4 ± 0.8 b	3.4 ± 0.1 a	11.7 ± 1.5 b	10.4 ± 1.7 b	19	**
	EC	51.9 ± 0.7 a	60.5 ± 0.1 b	51.8 ± 2.0 a	57.6 ± 0.2 b	22	**
	ECG	17.5 ± 0.2 b	11.7 ± 0.3 a	12.5 ± 0.4 a	11.7 ± 0.3 a	222	***
Terminal units (%)	C	8.5 ± 0.1 a	10.3 ± 0.1 b	10.7 ± 0.2 b	8.0 ± 0.5 a	54	***
	EC	8.1 ± 0.02 c	9.1 ± 0.2 d	7.4 ± 0.08 b	6.2 ± 0.16 a	185	***
	ECG	4.2 ± 0.01 a	4.8 ± 0.05 b	5.4 ± 0.02 c	5.9 ± 0.02 d	1129	***
Flavan-3-ols (mg/g DW)	C	0.18 ± 0.02 a	0.50 ± 0.07 b	0.47 ± 0.03 b	0.20 ± 0.04 a	31	**
	EC	0.14 ± 0.00 a	0.38 ± 0.06 b	0.21 ± 0.02 a	0.17 ± 0.02 a	21	**

^1^ Different letters along the line discriminate the cultivars significantly different from one another (*p* < 0.05, Tukey’s test). ^2^ Significance: *, **, and *** represent significance at *p* ≤ 0.05, 0.01 and 0.001, respectively.

**Table 3:** Average polyphenolic composition of the grape seeds according to the sampling time (after 2 days of maceration and at racking off). Effect of sampling time and interactions between sampling time and cultivar (2-factors ANOVA). mDP = mean degree of polymerization; G% = percentage of galloylation; EGC = (−)-epigallocatechin; C = (+)-catechin; EC = (−)-epicatechin; ECG = (−)-epicatechin-3-O-gallate.

		2 Days	Racking off	F	Sig	Sig F_cv*time_
Total flavonoids (mg/g DW)		85.0	40.7	66	*** ^1^	***
Total polyphenols (GAE) (mg/g DW)		44.2	26.3	454	***	***
mDP		3.6	4.6	220	***	***
G%		18.2	18.4	4	ns	***
Condensed tannins (mg/g DW)		22.0	14.2	292	***	***
Extention units (%)	EGC	0.29	0.38	39	***	ns
	C	3.1	8.7	168	***	***
	EC	56.7	55.4	4.9	ns	***
	ECG	12.3	13.3	41	***	**
Terminal units (%)	C	11.3	9.3	65	***	**
	EC	10.3	7.7	182	***	***
	ECG	5.9	5.1	1303	***	***
Flavan-3-ols (mg/g DW)	C	1.00	0.34	162	***	**
	EC	0.64	0.23	133	***	*

^1^ Significance: *, **, *** and ns represent significance at *p* ≤ 0.05, 0.01, 0.001 and not significant, respectively.

**Table 4:** Average results of the antioxidant tests performed (a) after 2 days of maceration and (b) after racking off. ANOVA and Tukey’s test results. AAE: mg of ascorbic acid equivalent per 1 g of seed flour (1 g DW); TE: mmols of Trolox equivalent per 100 g DW; Fe^2+^: mmols Fe^2+^/100 g DW; EC_20_ (Efficient Concentration): Quantity in mg of seed flour (DW) necessary to determine a 20% inhibition to 1 mg of 2,2 diphenyl-1-picrylhydrazyl (DPPH).

		Barbera	Grignolino	Nebbiolo	Uvalino	F_cv_	Sig
a	ABTS_AAE_	43.9 a ^1^	79.6 c	67.8 b	92.5 d	318	*** ^2^
	ABTS_TE_	24.9 a	45.2 c	38.5 b	52.5 d	318	***
	DPPH_AAE_	22.6 a	41.4 b	40.2 b	52.7 c	60.7	***
	DPPH_TE_	12.8 a	23.5 b	22.8 b	29.9 c	60.7	***
	FRAP_AAE_	34.4 a	72.2 c	58.9 b	85.3 d	1242	***
	FRAP_TE_	22.4 a	47.0 c	38.4 b	55.6 d	1242	***
	FRAP_Fe2+_	41.5 a	87.0 c	51.1 b	102.9 d	1242	***
	EC_20_	1.08 c	0.40 a	0.64 b	0.40 a	487	***
b	ABTS_AAE_	37.8 a ^1^	57.8 b	33.9 a	35.8 a	97	*** ^2^
	ABTS_TE_	21.5 a	32.8 b	19.2 a	20.3 a	97	***
	DPPH_AAE_	18.6 a	30.9 c	24.8 b	26.5 b	78.6	***
	DPPH_TE_	10.6 a	17.6 c	14.1 b	15.1 b	78.6	***
	FRAP_AAE_	29.9 a	48.0 c	31.5 a	35.2 b	220	***
	FRAP_TE_	19.5 a	31.3 c	20.5 a	23.0 b	220	***
	FRAP_Fe2+_	36.0 a	57.9 c	38.0 a	42.5 b	220	***
	EC_20_	1.58 b	0.69 a	1.27 b	1.01 ab	14.8	**

^1^ Different letters along the line discriminate the cultivars that are significantly different from one another (*p* < 0.05, Tukey’s test). ^2^ Significance: **, and *** represent significance at *p* ≤ 0.01 and 0.001, respectively.

**Table 5:** Correlation matrix between the parameters describing the polyphenolic content (total flavonoids (TF), total polyphenols (TP) and condensed tannins (CT)), and the results of the antioxidant tests.

	TF	TP	CT	ABTS	DPPH	FRAP	EC_20_
TF	1						
TP	0.977	1					
CT	0.953	0.986	1				
ABTS	0.973	0.982	0.991	1			
DPPH	0.960	0.983	0.967	0.953	1		
FRAP	0.980	0.985	0.986	0.989	0.978	1	
EC_20_	−0.851	−0.908	−0.914	−0.895	−0.905	−0.904	1
